# PSMA-directed CAR-T cell therapy for metastatic castration-resistant prostate cancer: a next-generation engineering perspective on stem cell-derived immune effectors

**DOI:** 10.3389/fimmu.2026.1819701

**Published:** 2026-04-15

**Authors:** Meng Zhang, Huimin Li, Kaisen Liao

**Affiliations:** 1 Tongde Hospital of Zhejiang Province, Hangzhou, China; 2 Zhejiang Academy of Traditional Chinese Medicine, Hangzhou, China

**Keywords:** CAR-T cells, induced pluripotent stem cells, metastatic castration-resistant prostate cancer, PSMA, synthetic biology, translational medicine, tumor microenvironment

## Abstract

The clinical translation of prostate-specific membrane antigen (PSMA)-directed chimeric antigen receptor (CAR) T-cell therapy for metastatic castration-resistant prostate cancer (mCRPC) has reached a critical impasse. Despite compelling preclinical rationale and early biological activity, durable clinical responses remain scarce, constrained by three core solid tumor challenges: a profoundly immunosuppressive/metabolically hostile tumor microenvironment (TME), pervasive antigen heterogeneity driving immune escape, and intrinsic limitations in T-cell fitness and *in vivo* persistence. This review synthesizes the current translational landscape (updated to February 2026), and posits a tripartite synergistic framework to systematically deconstruct these barriers: (1) advances in CAR synthetic biology; (2) active TME reprogramming via armored CAR-T cells, stromal-targeting agents, and rational combinations; (3) next-generation cellular product paradigms, with a focus on stem cell-derived immune effectors. Emerging platforms, including induced pluripotent stem cell (iPSC)-derived CAR-T, CAR-natural killer (NK) cells, and CAR-macrophages, offer unprecedented opportunities to overcome autologous product limitations via off-the-shelf availability, enhanced persistence, and intrinsic TME resistance. We further delineate a translational roadmap emphasizing biomarker-driven adaptive trials, predictive humanized preclinical models, and accessibility strategies. All core claims are graded using the 2011 Oxford Centre for Evidence-Based Medicine (OCEBM) Levels of Evidence to ensure academic rigor. This work provides a strategic blueprint to advance PSMA-CAR-T therapy toward curative-intent mCRPC treatment, with insights broadly applicable to next-generation stem cell-derived immunotherapies.

## 1 Introduction

Prostate cancer endures as a leading cause of cancer-related mortality among men globally (1). The progression to metastatic castration-resistant prostate cancer (mCRPC) represents a lethal disease stage, characterized by resistance to androgen receptor (AR) pathway inhibitors and a dismal prognosis with limited curative options (2). While therapeutic advances including taxane chemotherapy, novel hormonal agents, poly(ADP-ribose) polymerase (PARP) inhibitors, and PSMA-targeted radioligand therapy (RLT) have expanded the treatment landscape, these interventions predominantly yield palliative rather than durable remissions, highlighting an urgent, unmet clinical need for transformative therapeutic strategies (2).

Adoptive cellular therapy, particularly CAR-T cell therapy, has catalyzed a paradigm shift in the management of hematologic malignancies (3). This approach involves the genetic redirection of autologous T cells to express synthetic receptors that confer specific cytotoxicity against tumor-associated antigens (3). The translation of this success to solid tumors, however, has been markedly more challenging, largely due to the absence of ideal target antigens and a hostile tumor ecosystem (4, 5). Prostate-specific membrane antigen (PSMA), a type II transmembrane glycoprotein encoded by the FOLH1 gene, emerges as a premier candidate (6). It exhibits overexpression by several orders of magnitude on prostate cancer cells, with further upregulation in mCRPC and metastatic lesions, while maintaining minimal expression in most vital normal tissues (7). This expression profile nominates PSMA as a compelling immunotherapeutic target for prostate cancer (6).

The arsenal of PSMA-targeted therapies is expanding, now including RLT ([¹^77^Lu]Lu-PSMA-617, Pluvicto) and bispecific T-cell engagers (e.g., acapatamab) (8). Within this spectrum, CAR-T cell therapy offers distinct theoretical advantages: potential for sustained *in vivo* clonal expansion, long-term persistence, and the establishment of immunologic memory, collectively positioning it as a potential one-time curative intervention (5). Yet, the translation of this potential into consistent clinical efficacy for mCRPC has been incremental, impeded by the complex pathobiology inherent to solid tumors.

The disparity between robust preclinical activity and modest clinical outcomes for PSMA-CAR-T therapy underscores persistent, interconnected translational barriers: a profoundly immunosuppressive TME, inadequate T-cell trafficking and intratumoral persistence, pervasive antigen heterogeneity and escape, and the risk of on-target, off-tumor toxicity (9). This review contends that overcoming this translational impasse requires a fundamental shift from singular optimizations to a systems-level, integrated strategic framework. Herein, we critically analyze the translational trajectory of PSMA-CAR-T cells, distill lessons from early clinical trials, and articulate a tripartite translational framework structured around three synergistic pillars: (I) advancing CAR synthetic biology, (II) reprogramming the immunosuppressive TME, and (III) innovating cellular product paradigms—with a specific emphasis on next-generation platforms that engineer stem cells into functional immune effectors. We subsequently extend this framework into an actionable developmental roadmap, focusing on predictive preclinical models and next-generation clinical trial designs. Our objective is to provide a coherent, strategic guide intended to propel PSMA-CAR-T therapy from its current investigational status toward a definitive role in the curative management paradigm for mCRPC, with insights applicable to the broader solid tumor immunotherapy field and the emerging era of stem cell-derived cellular therapies. This work fully aligns with the scope of the Research Topic “Next-Generation of CAR-T Cell Therapy in Hematologic Malignancies and Solid Tumors”. While recent reviews have broadly covered CAR-T therapy in prostate cancer, our work provides a uniquely focused and in-depth analysis of PSMA-directed CAR-T cells, with a detailed synthesis of the latest clinical trial data (updated to February 2026) and a dedicated, forward-looking framework centered on next-generation stem cell-derived engineering strategies.

### 1.1 Literature search strategy

A systematic literature search was performed in accordance with the Preferred Reporting Items for Systematic Reviews and Meta-Analyses (PRISMA) guidelines, using the PubMed, Web of Science, and [ClinicalTrials.gov](https://ClinicalTrials.gov) databases. To capture the foundational knowledge and historical context, our initial search was broad and not restricted by date, focusing on seminal works that established key concepts (e.g., PSMA biology, CAR-T cell fundamentals). Subsequently, to ensure comprehensive coverage of the rapidly evolving recent advances, we conducted a focused search for peer-reviewed articles published between January 2015 and December 2025 using the following key terms and their Boolean combinations: “metastatic castration-resistant prostate cancer”, “mCRPC”, “prostate-specific membrane antigen”, “PSMA”, “chimeric antigen receptor T cell”, “CAR-T”, “tumor immune microenvironment”, “immunosuppression”, “T cell exhaustion”, “combination immunotherapy”, “radioligand therapy”, “bispecific T-cell engager”, “translational medicine”, “induced pluripotent stem cells”, “iPSC”, “CAR-NK”, and “CAR-macrophage”. The search was limited to English-language publications. Titles, abstracts, and full texts were screened for relevance. We additionally verified the latest status of all included clinical trials via ClinicalTrials.gov (data cutoff: February 2026; accessed February 28, 2026). This narrative synthesis integrates findings from seminal preclinical studies, published clinical trial results, and high-impact reviews to provide a mechanistically grounded, translational perspective on the evolution and future directions of PSMA-CAR-T therapy. All core clinical and mechanistic claims are graded using the 2011 OCEBM Levels of Evidence to ensure academic rigor and reproducibility.

## 2 PSMA biology and CAR design evolution

### 2.1 PSMA as a therapeutic target: CAR-T-relevant biological rationale

PSMA is a 750-amino acid transmembrane glycoprotein with folate hydrolase and N-acetylated-α-linked-acidic dipeptidase (NAALADase) activities. Beyond its enzymatic functions in nutrient metabolism and neuropeptide signaling, PSMA is implicated in oncogenic processes central to prostate cancer progression, including activation of PI3K/Akt and MAPK signaling pathways, promotion of angiogenesis, and facilitation of metastasis (10) (OCEBM Level 4, preclinical mechanistic study). Its near-uniform, high-density expression on the surface of prostate adenocarcinoma cells provides an optimal configuration for efficient immune synapse formation and CAR-T cell-mediated cytotoxicity. The marked upregulation of PSMA in mCRPC, particularly in metastatic lesions (including the dominant bone metastatic sites), further enhances its attractiveness as a target for advanced disease (7) (OCEBM Level 3, retrospective tissue cohort study).

Critically, PSMA biology directly informs the core translational barriers of PSMA-CAR-T therapy: its intrinsic heterogeneous and plastic expression drives antigen escape under therapeutic selective pressure (9) (OCEBM Level 3, multi-cohort clinical correlative study), while low-level physiological expression in salivary glands, renal proximal tubules, and small bowel epithelium drives predictable on-target, off-tumor toxicity (11) (OCEBM Level 3, Phase I clinical trial correlative analysis), defining the core requirements for optimized CAR design.

### 2.2 Evolution of PSMA-targeted CAR architectures

CAR design for PSMA-directed therapy has undergone iterative refinement to enhance T-cell activation potency, functional durability, and *in vivo* persistence, with each generation directly addressing limitations of prior constructs in mCRPC applications. All design iterations are supported by preclinical and clinical evidence, with OCEBM evidence levels graded accordingly.

#### 2.2.1 First-generation CARs

These pioneering constructs fused an anti-PSMA single-chain variable fragment (scFv) directly to the intracellular CD3ζ signaling domain. While they established proof-of-concept for antigen-specific T-cell redirection in early trials (NCT01140373), they elicited limited T-cell proliferation and transient *in vivo* activity due to the absence of co-stimulatory signaling, leading to activation-induced cell death and poor therapeutic efficacy (12) (OCEBM Level 3, first-in-human Phase I trial).

#### 2.2.2 Second-generation CARs

The incorporation of a single co-stimulatory domain (e.g., CD28 or 4-1BB) in tandem with CD3ζ marked a pivotal advancement, and remains the backbone of all current clinical PSMA-CAR-T products. CD28-based signaling promotes rapid effector function, robust IL-2 production, and metabolic reprogramming toward glycolysis, but may be associated with a more differentiated, effector-like phenotype and potential for exhaustion. In contrast, 4-1BB signaling favors mitochondrial biogenesis, oxidative metabolism, and enhanced persistence, often yielding a memory-rich T-cell population with superior long-term activity in mCRPC clinical models (13) (OCEBM Level 4, preclinical mechanistic study). Beyond canonical domains, inducible T-cell co-stimulator (ICOS) co-stimulation has also been explored to enhance CAR-T persistence and anti-tumor activity in preclinical prostate cancer models (14) (OCEBM Level 4, preclinical study).

#### 2.2.3 Third-generation CARs

These constructs combine multiple co-stimulatory signals (e.g., CD28 + 4-1BB) within a single receptor, aiming to synergistically amplify T-cell activation and survival signals. While certain preclinical models demonstrate enhanced anti-tumor potency, the increased signaling complexity raises concerns about potential overstimulation, accelerated exhaustion, and a less predictable pharmacokinetic profile, necessitating careful optimization. To date, these constructs have not advanced to pivotal clinical testing for mCRPC.

#### 2.2.4 Next-generation CARs

The field is now moving towards highly engineered, multifunctional receptors. This includes logic-gated CARs (AND, OR, NOT), synthetic receptor systems (e.g., synNotch), and constructs with optimized intrinsic properties. A landmark 2025 study introduced a transformative design by integrating the pTα-1A domain from the pre-TCR into the CAR intracellular region. This modification does not alter signaling but potently enhances the translation efficiency of CAR mRNA via the RNA-binding protein YBX1. The resulting CAR-T cells exhibit dramatically increased proliferation, cytokine production, and persistence with reduced exhaustion, representing a breakthrough in engineering intrinsic T-cell fitness for solid tumors (15) (OCEBM Level 4, landmark preclinical mechanistic study).

In our view, while later-generation CARs offer sophisticated solutions to specific problems (e.g., logic-gating for specificity, pTα-1A for fitness), the second-generation 4-1BBζ or CD28ζ backbone remains the most clinically validated platform for PSMA-CAR-T therapy, with all published mCRPC clinical trial data to date derived from second-generation constructs. The choice of co-stimulatory domain is paramount, as it dictates the metabolic and differentiation trajectory of the T cells. The field’s future likely lies not in simply adding more signaling domains, but in strategically integrating a refined backbone with highly specific, next-generation features (such as pTα-1A or logic gates) to address the unique challenges of mCRPC, a strategy that is beginning to be evaluated in trials like NCT06046040.

### 2.3 Antigen selection and scFv optimization

While PSMA remains the primary target for prostate cancer immunotherapy, the challenge of antigen escape necessitates exploring multi-targeting strategies. Ongoing trials such as NCT05437341, the first logic-gated bispecific CAR-T for mCRPC to enter clinical testing, are evaluating PSMA/CD70 bispecific CAR-T cells, while other prostate cancer-associated antigens including PSCA and STEAP1 continue to be investigated as alternative or complementary targets (16). Irrespective of the target antigen, the single-chain variable fragment (scFv) serves as the critical antigen-sensing domain of the CAR. The affinity of the scFv requires careful optimization: excessively high affinity may increase the risk of on-target, off-tumor toxicity and induce tonic signaling that predisposes T cells to exhaustion; conversely, insufficient affinity may result in suboptimal tumor recognition and activation. Emerging clinical data from PSMA-targeted bispecific T-cell engagers further underscore the importance of fine-tuning antigen-binding affinity to balance efficacy and toxicity (8). Beyond affinity, the scFv format (VH-VL orientation), linker design, and overall stability are critical determinants of proper CAR expression, immune synapse formation, and functional avidity. Emerging engineering strategies include the use of affinity-tuned scFvs and the adoption of nanobody (VHH)-based antigen recognition domains, which offer advantages in stability and tissue penetration. Optimizing these parameters in the context of PSMA’s unique expression profile and the immunosuppressive mCRPC TME is essential for achieving a favorable therapeutic window.

## 3 Clinical translation: early lessons and persistent gaps

### 3.1 Synopsis of emerging clinical data

Initial Phase I trials have unequivocally demonstrated the feasibility of manufacturing and administering autologous PSMA-CAR-T cells to patients with heavily pretreated mCRPC, establishing an acceptable initial safety profile. Subsets of patients have shown hallmarks of biological activity, including PSA reductions ≥30% and declining circulating tumor cell counts, indicating PSMA target engagement, though durable clinical efficacy remains unproven in the absence of a control arm. However, objective radiographic response rates (by Response Evaluation Criteria in Solid Tumors version 1.1 [RECIST 1.1] or Prostate Cancer Working Group 3 [PCWG3] criteria) remain modest, typically below 30%, and durable complete responses are exceedingly rare. We synthesized the latest clinical trial data (status updated to February 2026) to identify actionable trends, with full trial details, evidence grading, and translational insights summarized in **Table 1**.

**Table 1:** Selected clinical trials of PSMA-targeted CAR-T strategies in mCRPC (status updated to february 2026, with OCEBM evidence grading).

NCT identifier & phase	Strategy class	Target antigen (s)	Intervention	Status (last update)	Key findings & insights	OCEBM evidence level
NCT01140373 (Phase 1)	First-Gen CAR-T	PSMA	First-gen anti-PSMA CAR-T + cyclophosphamide/IL-2	Active, not recruiting (2026-01-15, long-term follow-up)	Historic proof-of-concept; biological activity (PSA declines) observed in enrolled patients (n=13) (12)	Level 3
NCT03089203 (Phase 1)	Armored CAR-T	PSMA	CART-PSMA-TGFβRDN (PSMA-CAR + dnTGFβRII)	Active, not recruiting (2026-02-23)	Published: Feasibility and safety established. Biological activity (PSA decline ≥50% in 4/13 pts) observed. Notable immune-mediated toxicities (CRS, including one fatal grade 4 event) occurred. Insight: Validates TGF-β armoring but underscores the challenge of balancing potency with toxicity (17)	Level 3
NCT04227275 (Phase 1)	Armored CAR-T	PSMA	CART-PSMA-TGFβRDN + lymphodepletion	Terminated (2023-08-21)	Termination Reason: “Based on the safety events and evidence of biologic activity without sustained clinical responses the Sponsor finds the risk/benefit analysis unfavorable for patients.” Insight: Highlights the critical need for better conditioning regimens, patient selection, and strategies to sustain responses	Level 3
NCT03873805 (Phase 1)	Alternative Target CAR-T	PSCA	Anti-PSCA-CAR-4-1BBζ T cells	Active, not recruiting (2025-06-26)	Published: Safety established. PSA declines >30% in 4/14 pts, with one durable response >28 days. Limited CAR-T persistence beyond 28 days noted. Insight: Explores PSCA as an alternative target; limited persistence highlights need for enhanced T-cell fitness (18)	Level 3
NCT05437341 (Phase 1/2)	Bispecific CAR-T	PSMA & CD70	PSMA/CD70 bispecific CAR-T cells	Recruiting (2022-06-29)	No published results to date. Insight: Dual-targeting strategy against antigen escape; CD70 co-engagement may also provide intrinsic co-stimulation (19)	Level 4 (preclinical rationale)
NCT06046040 (Phase 1)	Next-Gen Optimized CAR-T	PSMA	TmPSMA-02 CAR T cells (novel construct)	Recruiting (2025-12-22)	No published results to date. Preliminary safety/efficacy data expected after 2027. Insight: Tests an optimized next-generation CAR construct in an ongoing dose-escalation study	Level 4 (preclinical rationale)

CAR-T, chimeric antigen receptor T-cell; TGF-βRII, transforming growth factor-beta receptor II; dn, dominant-negative; CRS, cytokine release syndrome; PSA, prostate-specific antigen; pts, patients; OCEBM, Oxford Centre for Evidence-Based Medicine; PSCA, prostate stem cell antigen.

Trial status information is sourced from [ClinicalTrials.gov](https://ClinicalTrials.gov), and is current as of the “Last Update Posted” date indicated for each entry (data cut-off: February 2026; accessed February 28, 2026).

The emerging toxicity profile is dominated by cytokine release syndrome (CRS), which is typically low to moderate in grade and manageable with standard supportive care including tocilizumab. A predictable on-target, off-tumor toxicity is xerostomia, attributable to physiologically low-level PSMA expression in salivary gland acinar and ductal cells (11) (OCEBM Level 3, Phase I trial). Quantitative data from the seminal Phase I trial of TGF-β-insensitive armored PSMA-CAR-T cells (CART-PSMA-TGFBRDN, NCT03089203, total enrollment 23 patients, latest update February 2026) crystallize the central challenge: among 13 evaluable patients, 4 (31%) achieved a PSA decline ≥50%, but 5 patients (38%) experienced grade ≥2 CRS, including one fatal grade 4 CRS complicated by Enterococcus sepsis, occurring in the context of massive clonal CAR-T expansion (147, 019 copies/μg DNA peak) (17) (OCEBM Level 3, Phase I clinical trial). This underscores the intrinsic link between potent T-cell activation (necessary for efficacy) and the risk of significant immune-mediated toxicity—a “class effect” also prominently observed with PSMA-targeted bispecific T-cell engagers (BiTEs), where CRS rates can exceed 90% (8) (OCEBM Level 3, Phase I dose-escalation trial). Additional investigational products such as P-PSMA-101 have also demonstrated preliminary biological activity in Phase I testing, further validating the feasibility of PSMA-CAR-T therapy, while reinforcing the core challenge of balancing efficacy and toxicity (16) (OCEBM Level 3, Phase I trial). Thus, the paramount translational objective is to engineer strategies that widen the therapeutic window.

Additional published clinical data further define core limitations: the Phase I trial of PSCA-directed 4-1BB CAR-T cells (NCT03873805, enrollment 14 patients, published 2024) demonstrated PSA declines >30% in 4 of 14 patients, but limited CAR-T persistence beyond 28 days was universally observed, directly correlating with short duration of response (18) (OCEBM Level 3, Phase I clinical trial). A multi-center Phase I trial of the same CART-PSMA-TGFBRDN construct (NCT04227275, enrollment 16 patients) was terminated in 2023, with the sponsor citing “unfavorable risk/benefit analysis based on safety events and evidence of biologic activity without sustained clinical responses”, highlighting the critical need for optimized patient selection and combination strategies to sustain long-term responses.

#### 3.1.1 Safety profile refinement: beyond CRS to ICANS

In addition to CRS, immune effector cell-associated neurotoxicity syndrome (ICANS) is a recognized, potentially severe toxicity of CAR-T therapy, with standardized grading and management established by ASTCT consensus criteria (20) (OCEBM Level 3, consensus guideline). The incidence and severity of ICANS vary substantially across different CAR-T constructs and hematologic malignancies. Critically, the observation that potent PSMA engagement can drive neurotoxicity—first established in studies of PSMA-targeted bispecific T-cell engagers (BiTEs) (8)—has emerged as a pivotal safety consideration and is now a central hypothesis under investigation in the PSMA-CAR-T context. In contrast, emerging data specific to solid tumor CAR-T therapies, including those for prostate cancer, suggest a more favorable neurotoxicity profile. Early-phase CAR-T trials for prostate cancer (e.g., targeting PSCA) report ICANS as an infrequent (0-15%) and typically low-grade (grade 1-2) event. This reduced incidence may reflect lower baseline tumor burden and distinct cytokine release patterns compared to hematologic malignancies. Thus, while the risk of neurotoxicity must be vigilantly managed, it may constitute a less formidable barrier in the development of PSMA-CAR-T therapy for mCRPC.

#### 3.1.2 The promise of rational combinations: learning from PSMA-targeted therapies

The limited single-agent activity of current PSMA-CAR-T constructs underscores the necessity for rational combination strategies. Insights can be drawn from other PSMA-targeted modalities. The landmark VISION trial established [¹^77^Lu]Lu-PSMA-617 (Pluvicto) as a standard of care, demonstrating a significant improvement in median overall survival (mOS: 15.3 vs. 11.3 months) and radiographic progression-free survival (rPFS: 8.7 vs. 3.4 months) over standard of care in post-chemotherapy mCRPC (21) (OCEBM Level 1, randomized controlled trial). This RLT induces immunogenic cell death, potentially remodeling the TME. Early-phase trials combining a single dose of [¹^77^Lu]Lu-PSMA-617 with the PD-1 inhibitor pembrolizumab have demonstrated safety and provided mechanistic insights. Critical multi-omic analyses from the PRINCE trial reveal that treatment sequencing is vital. Administering RLT before or concurrently with PD-1 blockade leads to more favorable immune profiles—including stable T-cell levels and increased dendritic cells—compared to initiating immunotherapy first. This provides a strong rationale for “RLT priming” before CAR-T cell infusion, a strategy aimed at debulking tumor, inducing pro-inflammatory changes, and potentially enhancing CAR-T cell recruitment and function (22) (OCEBM Level 2, Phase I/II dose-expansion trial).

#### 3.1.3 Latest insights from combination trials

Recent clinical data continue to highlight the potential of combination approaches. The seminal phase I trial of TGF-β-insensitive (armored) PSMA-CAR-T cells demonstrated that 4 of 13 evaluable patients (31%) achieved a PSA decline ≥50%, providing proof-of-concept for biological activity (17). Furthermore, early-phase studies exploring combinations of PSMA-CAR-T with PD-1 inhibitors have reported encouraging preliminary signals, including observed objective responses and evidence of CAR-T persistence beyond six months in some patients. These findings collectively reinforce the hypothesis that overcoming the immunosuppressive TME is critical for unlocking the full potential of CAR-T therapy in mCRPC. An overview of clinical trials exploring these and other strategies is presented in **Table 1**.

### 3.2 Deconstructing the translational gaps

The constrained clinical efficacy, despite clear evidence of target engagement, can be attributed to a convergence of interrelated biological barriers that constitute the “solid tumor challenge”, with direct supporting evidence from mCRPC clinical trials.

#### 3.2.1 A Dominantly immunosuppressive TME

The prostate cancer TME is replete with soluble immunosuppressive mediators (e.g., TGF-β, IL-10, adenosine, prostaglandin E2) and a rich infiltrate of regulatory immune cells (regulatory T cells [Tregs], myeloid-derived suppressor cells [MDSCs], tumor-associated macrophages [TAMs]). These elements collectively induce CAR-T cell dysfunction, anergy, and premature apoptotic death, directly limiting expansion and cytolytic capacity in situ (23) (OCEBM Level 3, retrospective tissue cohort study). Furthermore, the stromal compartment, particularly cancer-associated fibroblasts (CAFs), actively shapes this suppressive milieu through mechanisms such as NNMT-driven metabolic reprogramming and extracellular matrix remodeling (24) (OCEBM Level 4, landmark preclinical study). Even with TGF-β armoring, >60% of patients treated with CART-PSMA-TGFBRDN did not achieve a PSA ≥50% response, demonstrating that multi-modal TME modulation is required to overcome redundant immunosuppressive pathways (17) (OCEBM Level 3, Phase I trial). Critically, the bone metastatic TME—the dominant site of mCRPC spread—exhibits a uniquely immunosuppressive phenotype, with osteoclast-osteoblast crosstalk further amplifying TGF-β signaling and myeloid cell infiltration, creating an immune-excluded niche that is particularly refractory to CAR-T infiltration (25) (OCEBM Level 3, preclinical mechanistic study).

#### 3.2.2 Physiologic and metabolic exclusion

Solid tumors present formidable physical barriers, including aberrant, non-productive vasculature and dense, fibrotic stroma that impede efficient CAR-T cell extravasation and infiltration. Furthermore, the intratumoral milieu is often nutrient-depleted (e.g., low glucose, oxygen) and acidic, creating a metabolically hostile environment that compromises T-cell survival, proliferation, and effector function (26) (OCEBM Level 3, preclinical mechanistic studies). In mCRPC bone metastases, the mineralized bone matrix adds an additional physical barrier, while the high metabolic demand of tumor cells in the bone niche exacerbates nutrient depletion and metabolic competition with infiltrating CAR-T cells.

#### 3.2.3 Dynamic antigen escape

PSMA expression is intrinsically heterogeneous and phenotypically plastic. Under the selective pressure of CAR-T cell therapy, tumor cell clones with low or negative PSMA expression possess a definitive survival advantage, culminating in antigen-negative relapse—a major mechanism of therapeutic failure observed across multiple CAR-T platforms (9) (OCEBM Level 3, multi-cohort clinical correlative study). Clinical data from PSMA-targeted therapies demonstrate that ~30% of progressing patients have evidence of PSMA downregulation or loss, making antigen escape a primary mechanism of treatment failure. Heterogeneous PSMA expression is particularly prevalent in bone metastatic lesions, further driving clonal selection and therapeutic resistance. This challenge has been extensively reviewed in the context of CAR-T resistance mechanisms (27) (OCEBM Level 3, systematic review).

#### 3.2.4 CAR-T cell functional exhaustion and limited persistence

Persistent antigen exposure within the immunosuppressive TME drives CAR-T cells toward a state of terminal dysfunction or exhaustion. This state is characterized by sustained expression of multiple inhibitory receptors (e.g., PD-1, LAG-3, TIM-3), epigenetic reprogramming, metabolic insufficiency, and loss of proliferative and effector potential, ultimately abrogating long-term tumor control (28) (OCEBM Level 4, preclinical mechanistic study). The molecular and cellular underpinnings of T cell exhaustion have been comprehensively defined (29) (OCEBM Level 3, systematic review). Clinical data directly link limited CAR-T persistence (<28 days) to short duration of response and lack of durable remission, as seen in the PSCA-CAR-T trial (NCT03873805) (18) (OCEBM Level 3, Phase I trial). This challenge is amplified in heavily pretreated mCRPC patients, whose autologous T cells often exhibit pre-existing functional impairment and senescence due to prior lines of chemotherapy and hormonal therapy.

## 4 A tripartite synergistic translational framework for optimization

**Figure 1:**
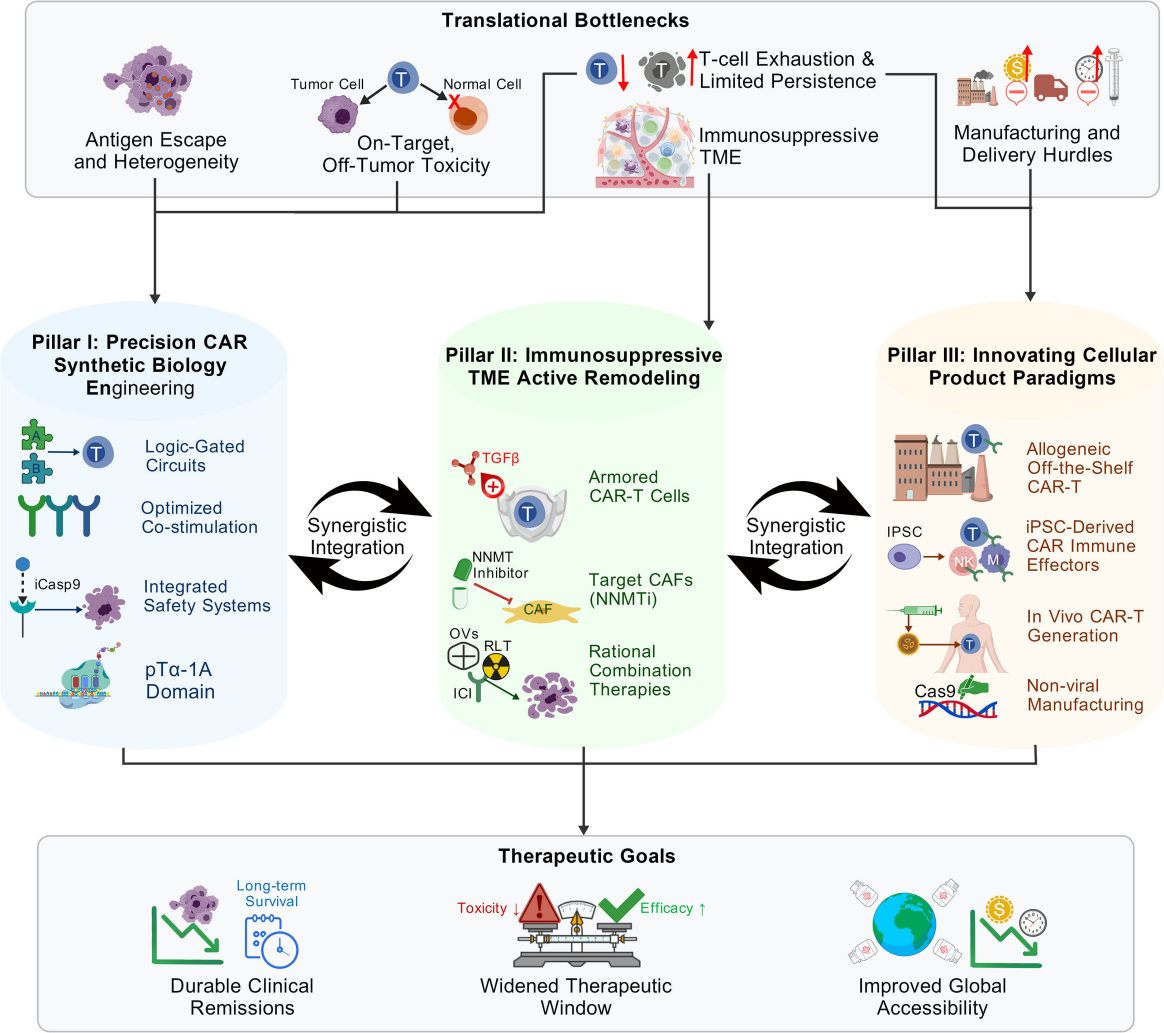
A tripartite synergistic translational framework to overcome core barriers of PSMA-targeted CAR-T therapy for metastatic castration-resistant prostate cancer (mCRPC). This schematic illustrates the integrated strategy to address the core translational bottlenecks of PSMA-CAR-T therapy (top panel) via three synergistic strategic pillars (middle panel), which collectively converge to the ultimate therapeutic goals (bottom panel). The top panel defines the five interrelated biological and logistical barriers limiting clinical efficacy, ordered from left to right: antigen escape and heterogeneity, on-target off-tumor toxicity, T-cell exhaustion and limited *in vivo* persistence, immunosuppressive tumor microenvironment (TME), and manufacturing and delivery hurdles. The middle panel delineates the three interdependent pillars: Pillar I focuses on precision CAR synthetic biology engineering to enhance antigen specificity, T-cell fitness and safety via logic-gated circuits, optimized co-stimulation, integrated safety control systems, and pTα-1A domain-mediated fitness enhancement; Pillar II centers on active remodeling of the immunosuppressive TME via TGF-β-insensitive armored CAR-T cells, NNMT inhibitor (NNMTi)-mediated cancer-associated fibroblast (CAF) targeting, and rational combination therapies including radioligand therapy (RLT), immune checkpoint inhibitors (ICIs) and oncolytic viruses (OVs); Pillar III innovates cellular product paradigms, ordered from top to bottom: allogeneic “off-the-shelf” CAR-T products, induced pluripotent stem cell (iPSC)-derived CAR immune effectors (CAR-T/CAR-NK/CAR-M), *in vivo* CAR-T generation, and non-viral manufacturing processes. Bidirectional arrows between the pillars highlight the synergistic integration of the three strategies. The bottom panel summarizes the final therapeutic outcomes enabled by this framework: durable clinical remissions, a widened therapeutic window balancing efficacy and toxicity, and improved global accessibility of cellular immunotherapy for mCRPC. CAR, chimeric antigen receptor; PSMA, prostate-specific membrane antigen; mCRPC, metastatic castration-resistant prostate cancer; TME, tumor microenvironment; TGF-β, transforming growth factor beta; CAF, cancer-associated fibroblast; NNMTi, nicotinamide N-methyltransferase inhibitor; RLT, radioligand therapy; ICI, immune checkpoint inhibitor; OV, oncolytic virus; iPSC, induced pluripotent stem cell; NK, natural killer.

To systematically dismantle the interconnected barriers delineated above, we propose an integrated translational framework structured upon three interdependent and synergistic pillars (**Figure 1**): (I) Precision CAR Construct Engineering via advanced synthetic biology, (II) Active TME Reprogramming, and (III) Next-Generation Cellular Product Paradigm Innovation. A multi-axis, combinatorial attack across these fronts is essential to convert transient biological activity into durable clinical cures. The strategic mapping of these barriers to integrated solutions, with corresponding OCEBM evidence grading, is summarized in **Table 2**.

**Table 2:** Strategic mapping of translational barriers to integrated solutions (with OCEBM 2011 evidence grading).

Translational barrier	Strategic approach	Exemplary tactics & clinical evidence	OCEBM evidence level	Current stage & key insight
Immunosuppressive TME	Pillar II: Remodeling (Armoring CAR-T Cells, Rational Combinations, Targeting Stromal Components)	• dnTGF-βRII armored CAR-T (CART-PSMA-TGFBRDN, NCT03089203) (17) • Combination with anti-PD-1/PD-L1 antibodies (30) • Sequencing with [¹^77^Lu]Lu-PSMA-617 RLT (22) • NNMT inhibitors: Targeting CAF-mediated immunosuppression (24) • Co-administration with oncolytic viruses (31)	Level 3 (clinical); Level 4 (preclinical)	Phase I/Translational: Armoring validated; highlights CRS management challenge. RLT + PD-1 combination shows sequencing is critical. NNMTi represents a promising stromal-targeting adjunct.
Antigen Escape & Heterogeneity	Pillar I: Engineering (Enhanced Specificity & Multi-Targeting, Alternative Antigens)	• Bispecific CARs (e.g., PSMA & PSCA or CD70, NCT05437341) (19) • Logic-gated (AND/OR/NOT) CAR circuits (19) • PSCA-directed CAR-T cells (NCT03873805) (18)	Level 3 (clinical); Level 4 (preclinical)	Phase I/II (Level 3 Evidence - Exploratory): Aims to prevent antigen-low relapse. Preclinical/Translational: AND-gate CARs enhance tumor specificity; first-in-human trials for prostate cancer initiating.
T-cell Exhaustion & Poor Persistence	Pillar I/III (Optimizing Intrinsic Fitness & Signaling, Manufacturing Innovations)	• Incorporation of 4-1BB or OX40 domains (13) • pTα-1A domain integration: Boosts mRNA translation and T-cell expansion (15) • Product manufacture from less-differentiated Tscm or naive T-cell subsets (32) • Engineering co-stimulatory ligand expression (e.g., GITRL)	Level 3 (clinical correlation); Level 4 (preclinical)	Translational/Breakthrough: Co-stimulation domain critically dictates metabolic fitness. pTα-1A is a novel, potent enhancer of CAR-T cell proliferative capacity and persistence. Cell subset composition (e.g., Tscm selection) remains a key determinant.
On-target, Off-tumor Toxicity	Pillar I: Engineering (Integrated Safety Systems)	• Inducible suicide genes (iCasp9) (33) • Truncated EGFR (tEGFR) marker/suicide switch • Affinity-tuned or logic-gated scFvs (19)	Level 3 (clinical validation)	Phase I (Level 3 - Safety Backstop): Essential for mitigating severe toxicity (e.g., high-grade CRS). Introduces trade-offs in immunogenicity and manufacturing.
Manufacturing & Delivery Hurdles	Pillar III: Innovation (Next-Generation Platforms, Process Innovation)	• Allogeneic (“off-the-shelf”) CAR-T from healthy donors (34) • *In vivo* CAR-T generation via targeted LNPs or viral vectors (35–37) • iPSC-derived CAR immune effectors (CAR-T, CAR-NK, CAR-M) (38–41) • Non-viral gene delivery systems (e.g., transposon/CRISPR) to streamline manufacturing	Level 3 (clinical proof-of-concept); Level 4 (preclinical)	Disruptive Technology Frontier: Aims to solve scalability, cost, and immediacy. iPSC-derived effectors represent a transformative off-the-shelf platform with multi-feature engineering capability. Non-viral methods are key to reducing complexity and cost.

CAR-T, chimeric antigen receptor T-cell; TGF-βRII, transforming growth factor-beta receptor II; dn, dominant-negative; CRS, cytokine release syndrome; RLT, radioligand therapy; TME, tumor microenvironment; PSMA, prostate-specific membrane antigen; OCEBM, Oxford Centre for Evidence-Based Medicine; iPSC, induced pluripotent stem cell; Tscm, stem cell memory T cell; NK, natural killer; LNP, lipid nanoparticle.

Bidirectional arrows between the pillars emphasize the necessity for synergistic integration: Pillar I enhances T-cell intrinsic fitness, antigen specificity, and safety, enabling improved function in the hostile TME addressed by Pillar II; Pillar III overcomes the biological and logistic limitations of autologous products, amplifying the efficacy of optimizations in Pillar I and II. Their combined output converges to drive the field toward the ultimate therapeutic goals: durable clinical remissions, a widened therapeutic window, and improved global accessibility of cellular immunotherapy for mCRPC.

### 4.1 Pillar I: precision CAR construct engineering via advanced synthetic biology

This pillar focuses on intrinsically enhancing the specificity, safety, controllability, and fitness of the CAR-T cell product through rational molecular design, directly addressing antigen escape, on-target off-tumor toxicity, and T-cell exhaustion.

#### 4.1.1 Refined signaling and logic-gated circuits

Concurrently, the exploration of novel signaling molecules (e.g., ICOS, OX40, CD27) and their stoichiometric combinations continues to refine T-cell phenotypes (42) (OCEBM Level 4, preclinical mechanistic study). The choice between CD28 and 4-1BB co-stimulation remains pivotal, imparting distinct metabolic and differentiation profiles. Furthermore, synthetic biology enables logic-gated CAR systems. AND-gate CARs require co-engagement of two tumor-associated antigens for full activation, conferring exquisite tumor specificity. The field is advancing to clinical testing, with the first programmable logic-gated CAR-T for mCRPC entering trials (NCT05437341, PSMA/CD70 bispecific CAR). NOT-gate or inhibitory CAR (iCAR) systems add another safety layer by suppressing activation upon recognition of a normal tissue antigen (19) (OCEBM Level 4, preclinical proof-of-concept study). The clinical translation of these multi-component systems demands rigorous evaluation of antigen pair biology and manufacturing feasibility.

#### 4.1.2 Integrated safety and control systems

To preemptively manage severe adverse events, fail-safe mechanisms are engineered into CAR-T cells, including the investigational TmPSMA-02 product (NCT06046040). Inducible suicide genes, such as rapamycin-activated caspase-9 (iCasp9), allow for the rapid, pharmacologically triggered elimination of the entire CAR-T cell population (33) (OCEBM Level 3, clinical validation in hematologic malignancies). Surface-expressed markers like truncated EGFR (tEGFR) serve a dual purpose: enabling *in vivo* tracking and providing a target for antibody-mediated depletion (e.g., using cetuximab). While clinically validated, these systems introduce additional genetic payload and complexity, necessitating a balanced design philosophy.

#### 4.1.3 Next-generation intrinsic engineering for enhanced fitness

Beyond optimizing co-stimulatory domains, frontier research is reprogramming the fundamental biology of CAR-T cells. A landmark 2025 study introduced a paradigm-shifting strategy: integrating a 48-amino acid domain, pTα-1A, derived from the pre-T cell receptor (TCR) α-chain, into the CAR’s intracellular region (between CD28 and CD3ζ) (15). This modification does not create a new signaling pathway but potently enhances the translation efficiency of the CAR mRNA. Mechanistically, the pTα-1A domain harbors a unique nucleotide sequence that forms a specific RNA secondary structure. This structure acts as a high-affinity binding platform for the ubiquitous RNA-binding protein YBX1 (Y-box binding protein 1). Upon recruitment, YBX1 stabilizes the CAR mRNA, facilitates its polysome loading, and thereby boosts *de novo* CAR protein synthesis. Quantitatively, CAR-T cells engineered with the pTα-1A domain demonstrated a 3- to 5-fold increase in CAR surface expression compared to conventional CAR-T cells, directly linking enhanced translation to functional output. Consequently, in multiple solid tumor models, including prostate cancer, CAR-T cells harboring this “19-28z-1A” construct exhibited markedly increased proliferation, heightened cytokine production, superior persistence, and reduced expression of exhaustion markers. When combined with other potency-enhancing mutations (e.g., “1XX” in the CD3ζ domain), it generated one of the most potent anti-solid tumor CAR-T products reported to date. This represents a breakthrough in engineering intrinsic T-cell fitness by harnessing post-transcriptional control (15) (OCEBM Level 4, landmark preclinical study).

### 4.2 Pillar II: active remodeling of the immunosuppressive TME

This pillar employs combination strategies and genetic “armoring” to convert the hostile mCRPC TME into a permissive and supportive milieu for CAR-T cell function, directly addressing the immunosuppressive and physical barriers to efficacy.

#### 4.2.1 Armored CAR-T cells

Genetic modification of CAR-T cells to resist dominant immunosuppressive signals remains a core strategy. Co-expression of a dominant-negative TGF-β receptor (dnTGFβRII) renders cells functionally insensitive to this key immunosuppressive cytokine, preserving their proliferation and cytotoxicity in the TME—an approach validated in preclinical prostate cancer models (43) (OCEBM Level 4, preclinical study) and the CART-PSMA-TGFBRDN clinical trial (17) (OCEBM Level 3, Phase I trial). This intrinsic armoring strategy is one of the most mature genetic engineering approaches to overcome the suppressive TME in solid tumors (44) (OCEBM Level 3, systematic review). Alternative approaches include engineering CAR-T cells to secrete PD-1-blocking scFv fragments or express a PD-1/CD28 switch receptor, enabling localized reversal of T-cell exhaustion without systemic checkpoint inhibitor exposure (45) (OCEBM Level 4, preclinical study). Additionally, arming CAR-T cells with pro-inflammatory cytokines such as IL-15 or IL-18 has emerged as a complementary strategy to enhance intrinsic T-cell fitness, promote resistance to exhaustion, and recruit endogenous immune cells to the tumor microenvironment. These “armored” CAR-T cells can deliver immunostimulatory payloads directly at the tumor site, remodeling the suppressive TME while mitigating systemic toxicity associated with cytokine administration (46, 47) (OCEBM Level 4, preclinical and review).

#### 4.2.2 Targeting stromal-immune crosstalk

The stromal compartment, particularly CAFs, is a master regulator of immunosuppression and a prime target for TME reprogramming. A 2025 Nature study identified nicotinamide N-methyltransferase (NNMT) as a critical metabolic checkpoint in CAFs. NNMT activity drives a pro-tumorigenic, immunosuppressive CAF phenotype via epigenetic rewiring (H3K27me3 hypomethylation), leading to complement-mediated recruitment of MDSCs. Pharmacological inhibition of NNMT reversed this phenotype, reduced MDSC infiltration, re-activated CD8+ T cells, and restored sensitivity to immune checkpoint blockade in preclinical models. This underscores that targeting CAF-derived metabolic pathways is a potent strategy for dismantling the immunosuppressive barriers of the TME (24) (OCEBM Level 4, landmark preclinical study). An orthogonal approach involves directly depleting CAFs or modulating their function via fibroblast activation protein (FAP)-targeted CAR-T cells, which has demonstrated the ability to reduce stromal barriers and enhance T-cell infiltration in preclinical solid tumor models (48) (OCEBM Level 4, preclinical and review). Beyond metabolism, targeting the physical and biochemical barrier posed by CAFs is equally critical. The insulin-like growth factor 1 receptor (IGF1R) pathway has been implicated in mediating stromal fibrosis and, more recently, identified as a key driver of T-cell dysfunction in prostate cancer. Research demonstrates that IGF-1 signaling through IGF1R can suppress antigen presentation and upregulate PD-L1, thereby facilitating immune evasion. Notably, this rationale has progressed to clinical testing, with an ongoing, early-phase combination trial (NCT06866548) currently recruiting patients; results are not yet available. This provides a strong translational rationale for exploring IGF1R inhibition as a strategy to modulate the fibrotic and immunosuppressive prostate TME, potentially in combination with CAR-T therapy (49) (OCEBM Level 4, preclinical mechanistic study).

#### 4.2.3 Rational combination therapies

Systemic combination with TME-modulating modalities is critical for transforming “cold” prostate tumors into immunologically “hot” lesions:

##### 4.2.3.1 Immune checkpoint inhibitors

Co-administration of anti-PD-1/PD-L1 antibodies may reverse CAR-induced or pre-existing T-cell exhaustion and counteract extrinsic suppression mediated by the TME (30) (OCEBM Level 4, preclinical study). Clinical data supports the synergistic potential of this combination in early-phase testing.

##### 4.2.3.2 PSMA-targeted radioligand therapy

[¹^77^Lu]Lu-PSMA-617 induces immunogenic cell death, promotes tumor debulking, upregulates PSMA expression, and disrupts fibrotic stroma to enhance CAR-T infiltration (50) (OCEBM Level 3, clinical guideline and multi-center dosimetry study). This creates a potential synergistic sequence—”debulk and modulate” with RLT followed by “target and eliminate” with CAR-T cells. Clinical data has further demonstrated the safety and preliminary efficacy of [¹^77^Lu]Lu-PSMA-617 combined with PD-1 blockade in mCRPC, providing a clinical foundation for RLT-CAR-T combination strategies (22) (OCEBM Level 2, Phase I/II dose-expansion trial).

##### 4.2.3.3 Oncolytic viruses

Engineered to selectively replicate in tumor cells, OVs trigger immunogenic cell death, release tumor antigens and damage-associated molecular patterns (DAMPs), and stimulate a pro-inflammatory innate immune response, effectively turning “cold” tumors “hot” and enhancing CAR-T cell recruitment and activity (31) (OCEBM Level 4, preclinical study). OVs can also be engineered to express immunomodulatory cytokines or checkpoint inhibitors, further synergizing with CAR-T function in the TME.

### 4.3 Pillar III: next-generation product paradigm innovation

This pillar aims to overcome the significant logistic, economic, and biological limitations of current patient-specific (autologous) CAR-T products, addressing manufacturing hurdles and T-cell fitness limitations in heavily pretreated mCRPC patients.

While autologous PSMA-CAR-T products have established proof-of-concept clinical activity, their broader clinical and commercial impact is constrained by three inherent limitations. First, the variable T-cell fitness of heavily pretreated patients—who often have exhausted or senescent T cells due to prior lines of chemotherapy and hormonal therapy—compromises product potency and durability. Second, the personalized manufacturing paradigm is complex, costly, and requires prolonged vein-to-vein time, limiting patient access and global scalability. Third, the per-patient manufacturing process precludes the iterative, multi-feature engineering that is likely necessary to overcome the complex barriers of solid tumors. Allogeneic products, particularly those derived from induced pluripotent stem cells (iPSCs), offer a compelling alternative by decoupling product quality from patient health, enabling true ‘off-the-shelf’ availability, and providing a platform for standardized, multi-transgenic engineering. **Table 3** provides a direct comparison of autologous, allogeneic, and iPSC-derived CAR platforms in the context of mCRPC.

**Table 3:** Comparative analysis of autologous, allogeneic, and iPSC-derived CAR platforms for Mcrpc.

Core parameters	Autologous CAR-T	Healthy donor-derived allogeneic CAR-T	iPSC-derived CAR immune effectors
Starting Cell Source	Patient’s own peripheral T cells	Healthy donor peripheral T cells	Clonal master iPSC line (renewable)
Core Advantages for mCRPC	No alloimmune rejection; HLA-matched to the patient	Off-the-shelf availability; superior T-cell fitness from healthy donors; standardized batch manufacturing	True off-the-shelf scalability; unlimited renewable source; compatible with multi-feature genetic engineering; can generate CAR-T, CAR-NK, CAR-M multiple lineages
Critical Limitations for mCRPC	Variable T-cell fitness in heavily pretreated patients; long vein-to-vein time; high personalized manufacturing cost; limited multi-gene engineering feasibility	Risk of GvHD (requires TCR knockout); limited *in vivo* persistence due to host immune rejection; constrained engineering complexity	CAR-T lineage: requires TCR knockout to eliminate GvHD risk; CAR-NK/CAR-M lineages: no endogenous TCR, inherent absence of GvHD risk; long-term safety of clonal master lines requires full clinical validation
GvHD Risk	None	Low (with TCR/B2M gene editing), otherwise high	CAR-T: Low (with TCR knockout); CAR-NK/CAR-M: None
Clinical Validation in mCRPC	Phase I proof-of-concept (multiple trials, e.g., NCT03089203)	No published mCRPC clinical data to date	No published mCRPC clinical data to date; preclinical proof-of-concept in multiple solid tumor models
Manufacturing Complexity & Cost	High, personalized per-patient manufacturing	Moderate, batch-produced; lower per-dose cost at scale	Lowest, renewable master cell line enables large-scale batch production; minimal per-dose incremental cost
OCEBM Evidence Level	Level 3 (Phase I clinical data)	Level 4 (preclinical rationale; Level 3 validation in hematologic malignancies)	Level 4 (preclinical proof-of-concept)

A central theme of next-generation innovation is the engineering of stem cells—particularly induced pluripotent stem cells (iPSCs)—into functional immune effectors, enabling off-the-shelf availability, standardized quality, and enhanced therapeutic potency.

#### 4.3.1 Allogeneic (“off-the-shelf”) CAR-T cells

Derived from healthy donors, these products are gene-edited to disrupt the endogenous T-cell receptor (TCR) and β2-microglobulin (B2M) to prevent graft-versus-host disease (GvHD) and host rejection (34) (OCEBM Level 3, clinical proof-of-concept in hematologic malignancies). This strategy promises immediate availability, standardized quality, and potentially lower cost. Critically, allogeneic products from healthy donors avoid the T-cell fitness limitations of autologous products derived from heavily pretreated mCRPC patients, who often have functionally impaired T cells due to prior lines of chemotherapy and hormonal therapy. Advanced engineering strategies, including TALEN-edited inducible dual CAR systems with integrated safety switches, have further enhanced the specificity and safety profile of allogeneic products for solid tumor applications (51) (OCEBM Level 4, preclinical study). Early-phase trials for universal CAR-T platforms are underway, though challenges like limited persistence due to host immune clearance remain a focus of ongoing research. The unique hurdles of applying allogeneic CAR-T platforms to solid tumors, including enhanced risk of rejection and navigating a more complex TME, have been recently elaborated (52) (OCEBM Level 3, systematic review).

#### 4.3.2 *In vivo* CAR-T cell generation

This disruptive paradigm seeks to bypass ex vivo manufacturing entirely by using targeted lipid nanoparticles (LNPs) or viral vectors to deliver CAR genes directly to a patient’s T cells *in vivo* (36, 37, 53) (OCEBM Level 4, preclinical proof-of-concept study). Preclinical studies have demonstrated the feasibility of generating functional CAR-T cells *in situ*, which could enable repeat dosing, simplify logistics, and dramatically improve accessibility. While promising, challenges related to targeting specificity, transduction efficiency, and safety *in vivo* require extensive investigation (35) (OCEBM Level 4, preclinical study).

#### 4.3.3 Manufacturing process innovations

Advances in autologous manufacturing also impact efficacy and accessibility. This includes selecting optimal T-cell subsets (e.g., naïve or stem cell memory T cells [Tscm]) as starting material to enhance persistence (32) (OCEBM Level 3, clinical correlative study). Furthermore, the development of non-viral gene delivery systems (e.g., using transposon systems or CRISPR-Cas9 for targeted integration) is a major frontier. These methods aim to improve safety profiles and, crucially, reduce the cost and complexity of manufacturing compared to traditional viral vector approaches, representing a key technological step toward improving the scalability and global access to CAR-T therapy.

#### 4.3.4 iPSC-derived immune effectors: a platform for next-generation off-the-shelf CAR therapies

Beyond traditional allogeneic approaches, the use of induced pluripotent stem cells (iPSCs) as a renewable cell source represents a transformative paradigm for next-generation cellular immunotherapy. iPSCs can be genetically engineered, clonally expanded, and subsequently differentiated into various immune effector lineages, including T cells, natural killer (NK) cells, and macrophages, offering unprecedented opportunities for off-the-shelf CAR therapy with standardized potency and scalability.

Recent preclinical advances have demonstrated the feasibility and therapeutic potential of iPSC-derived CAR effectors in solid tumors. A landmark 2025 study reported the development of HER2-targeting iPSC-derived CAR-T cells engineered to overcome multiple barriers to solid tumor efficacy. These cells incorporated a suite of genetic modifications—including IL-7 receptor fusion, a TGF-β-IL-18 receptor switch, and CXCR2 expression—to enhance persistence, resistance to immunosuppression, and tumor trafficking, resulting in potent anti-tumor activity in preclinical models (38) (OCEBM Level 4, landmark preclinical study). This work illustrates how iPSC platforms enable complex, multi-feature engineering that would be challenging to implement in primary T cells.

Beyond T cells, iPSC-derived CAR-NK cells offer distinct advantages, including intrinsic resistance to exhaustion, favorable safety profiles (absence of GvHD risk), and natural cytotoxicity against tumor cells. A 2026 study introduced a programmable iPSC-derived CAR-NK vesicle platform (CAR-iNEV), demonstrating that extracellular vesicles derived from these cells can remodel the immune microenvironment and eradicate tumors in preclinical models. This cell-free approach leverages the potent anti-tumor properties of CAR-NK cells while potentially mitigating risks associated with live cell infusion (40) (OCEBM Level 4, preclinical study).

iPSC-derived CAR-macrophages (CAR-iMAC) represent an emerging frontier, particularly for solid tumors where myeloid cells naturally infiltrate the TME. A comprehensive 2026 review highlighted the unique capabilities of CAR-iMAC, including their ability to phagocytose tumor cells, present antigens, and remodel the TME—functions that complement the cytotoxic activity of T and NK cells (41) (OCEBM Level 4, systematic review). To address the challenge of CAR-iMAC potentially adopting an immunosuppressive M2 phenotype within the TME, a 2025 perspective proposed targeting RNA-binding proteins Roquin-1 and Regnase-1 via CRISPR-Cas9. Simultaneous knockout of these regulators could drive CAR-iMAC toward a sustained M1-like pro-inflammatory state, enhancing their phagocytic capacity and cytotoxic function (39) (OCEBM Level 4, preclinical perspective).

The convergence of iPSC technology with advanced gene editing thus enables the generation of precisely engineered, off-the-shelf CAR immune effectors with defined phenotypes and enhanced functionalities. As these platforms mature, they hold the potential to fundamentally disrupt the current autocentric manufacturing model, democratizing access to effective cellular immunotherapies for mCRPC and beyond.

## 5 A roadmap for clinical translation: overcoming systemic bottlenecks

Bridging the gap between strategic conception and clinical realization requires addressing systemic bottlenecks in preclinical modeling, clinical trial methodology, and real-world implementation.

### 5.1 Developing physiologically relevant preclinical models

Traditional immunodeficient xenograft models fail to recapitulate the critical interplay between human CAR-T cells, a human immune system, and patient-derived tumor biology, particularly the unique immunosuppressive microenvironment of bone metastases (the dominant site of mCRPC spread). This is a core driver of the “preclinical effective, clinical inactive” translational gap. We recommend two priority actions:

#### 5.1.1 Standardize bone metastatic mCRPC humanized patient-derived xenograft models

The field must prioritize development and public sharing of well-characterized humanized mouse models engrafted with patient-derived bone metastatic mCRPC xenografts, with a reconstituted human immune system. These models must faithfully recapitulate PSMA heterogeneity, the bone TME, and metastatic tropism, to enable rigorous preclinical validation of next-generation strategies before human trials (54) (OCEBM Level 3, systematic review of humanized model applications). Recent advancements in such humanized mouse platforms have markedly improved their fidelity in modeling the human tumor-immune interface, making them indispensable tools for preclinical immuno-oncology research, including the evaluation of CAR-T cell therapies (55) (OCEBM Level 3, systematic review).

#### 5.1.2 Implement “clinical trial in a mouse” avatar models

For individual patients, avatar models can be used to predict response to specific CAR-T constructs and combination regimens, enabling personalized therapy selection and hypothesis generation for correlative clinical studies.

Establishing collaborative, publicly accessible repositories of well-characterized mCRPC PDX models with annotated PSMA expression and genomic profiles should be a community-wide priority.

### 5.2 Designing next-generation clinical trials

Future trials must evolve beyond small, sequential, single-arm Phase I studies to efficiently navigate the combinatorial complexity of the proposed framework.

#### 5.2.1 Biomarker-enriched patient stratification

Trial entry should mandate comprehensive baseline profiling to enrich for patients most likely to benefit and enable correlative science. This includes:

- Quantitative PSMA-PET imaging: Using standardized metrics (e.g., SUVmax, PSMA tumor volume) and scoring systems (e.g., PSMA-RADS) to define “PSMA-high” disease (minimum threshold of standardized uptake value [SUV] ≥10, aligned with PSMA RLT benefit criteria) and assess spatial heterogeneity.
- Circulating tumor DNA (ctDNA) analysis: Assessing baseline tumor mutational burden, specific genomic alterations, and tracking molecular response via ctDNA clearance.
- Deep immunophenotyping: Profiling peripheral blood and, when feasible, serial tumor biopsies for immune cell subsets, checkpoint expression, and soluble factors.

#### 5.2.2 Adaptive platform trial (master protocol) designs

To efficiently test multiple CAR-T constructs, armoring strategies, and combination partners in parallel, the field should implement adaptive platform trials. A single master protocol can randomize patients to different experimental arms (e.g., different CAR co-stimulation domains, ± pTα-1A enhancement, ± TGF-β armoring, ± concurrent ICI or RLT). Pre-specified adaptive rules based on interim analyses allow for the early discontinuation of underperforming arms and the seamless expansion of promising ones, dramatically accelerating the therapeutic optimization cycle (56) (OCEBM Level 2, systematic review of platform trial methodology). This design is particularly well-suited for mCRPC, where multiple combinatorial strategies require clinical evaluation.

#### 5.2.3 Comprehensive translational endpoints

Moving beyond traditional endpoints (PSA, RECIST), trials must incorporate dynamic, mechanism-informed biomarkers. These include ctDNA clearance (molecular response, which is more predictive of long-term survival than PSA decline), high-dimensional immune monitoring (e.g., mass cytometry, single-cell RNA sequencing) to track CAR-T and endogenous immune cell dynamics, and novel functional imaging to assess intratumoral CAR-T cell activity. These endpoints are exploratory and hypothesis-generating.

### 5.3 Fostering translational consortia

Accelerating progress demands breaking down traditional silos between academia, industry, and regulators. Structured public-private translational consortia (modeled on the Prostate Cancer Foundation’s Cell Therapy Consortium) are needed to: standardize biomarker assays and preclinical models; establish shared biorepositories of clinical samples; facilitate data sharing across trials; and align on regulatory pathways for complex cellular products and combination therapies, thereby de-risking and streamlining the entire development pipeline (57) (OCEBM Level 3, systematic review of cellular therapy translational bottlenecks).

### 5.4 Addressing accessibility, cost, and implementation pathways

The transformative potential of CAR-T therapy will remain limited unless challenges of cost and equitable access are addressed. Strategic solutions must be integrated into the development roadmap:

- Technology-Driven Cost Reduction: Advancing allogeneic “off-the-shelf” and *in vivo* generation platforms holds the greatest promise for reducing costs through scalability and standardization. iPSC-derived platforms, in particular, offer the potential for truly scalable, batch-produced cellular products, dramatically lowering per-dose costs. The maturation of non-viral gene delivery systems is crucial for simplifying autologous manufacturing.
- Operational Efficiencies: Establishing regional or centralized manufacturing centers can achieve economies of scale. Developing robust cryopreservation and logistics networks extends product shelf-life and reach.
- Innovative Payment Models: Collaborating with payers to develop value-based agreements, outcomes-linked pricing, and risk-sharing models can improve affordability.
- Global Health Considerations: For low-resource settings, ultra-simplified, point-of-care manufacturing approaches and the development of lower-cost viral vectors or non-viral systems are essential long-term goals.

## 6 Discussion

PSMA-CAR-T therapy for mCRPC stands at a decisive translational crossroads. As this review has synthesized, overcoming the solid tumor barrier is not a singular challenge but a systems-level problem demanding an integrated solution. The tripartite translational framework—encompassing precision CAR engineering (Pillar I), active TME reprogramming (Pillar II), and next-generation product innovation (Pillar III)—provides a coherent blueprint to deconstruct the intertwined biological and logistical hurdles. By framing mCRPC as a prototypical model for solid tumor CAR-T development, this work provides broadly applicable principles for addressing the universal barriers of TME immunosuppression, antigen heterogeneity, T-cell exhaustion, and limited product accessibility.

The emergence of stem cell-derived immune effectors, particularly those generated from iPSCs, represents a paradigm shift within Pillar III. These platforms address fundamental limitations of autologous products—including T-cell fitness, manufacturing scalability, and cost—while enabling levels of genetic engineering that are challenging to achieve in primary cells. As highlighted in this review, iPSC-derived CAR-T cells engineered to overcome multiple solid tumor barriers (38), programmable CAR-NK vesicles capable of remodeling the TME (40), and CAR-macrophages with engineered persistence (39, 41) collectively illustrate the transformative potential of this approach. The convergence of iPSC technology with advances in synthetic biology and gene editing positions these next-generation platforms as central to the future of cellular immunotherapy for solid tumors.

### 6.1 Limitations

This review has several limitations that must be acknowledged: (1) we focus specifically on PSMA-directed CAR-T therapy, and do not comprehensively cover other PSMA-targeted modalities (bispecific T-cell engagers, RLT) or other prostate cancer-associated antigen targets (PSCA, STEAP1) in depth; (2) emerging technologies such as iPSC-derived CAR effectors and *in vivo* CAR-T generation have limited clinical data to date, with long-term safety and efficacy yet to be established; (3) we focus on clinical and preclinical data published through December 2025, with ongoing trials (including NCT05437341 and NCT06046040) yet to report full results.

### 6.2 Field controversies and future outlook

Critical unresolved questions remain in the field, which will shape the future trajectory of PSMA-CAR-T therapy for mCRPC. First, the optimal treatment line and sequencing strategy remains undefined: while current trials enroll heavily pretreated patients with limited treatment options, preclinical data suggests that earlier-line intervention (before T-cell fitness is compromised by prior therapies) may yield superior outcomes. Second, the balance between potency and toxicity remains a central challenge: TGF-β armoring enhances CAR-T function in the TME, but may increase the risk of high-grade CRS, highlighting the need for refined engineering strategies that uncouple effector function from systemic inflammation. Third, the clinical value of logic-gated and multi-targeted CARs remains to be validated: while these constructs address antigen escape, their increased manufacturing complexity and regulatory hurdles may limit widespread clinical adoption. Fourth, the role of allogeneic and iPSC-derived CAR products in mCRPC remains to be defined: while they address the limitations of autologous products, their long-term persistence, immunogenicity, and manufacturing consistency require rigorous evaluation in clinical trials.

Looking forward, the trajectory of PSMA-CAR-T therapy will be profoundly shaped by convergence with other disruptive technologies. The integration of artificial intelligence (AI) and machine learning with high-throughput experimental data holds promise for in silico prediction of optimal CAR signaling architectures, identification of novel tumor-specific antigen combinations for logic-gating, and the discovery of resistance mechanisms from integrated multi-omic datasets (58) (OCEBM Level 4, preclinical and translational review). Simultaneously, advances in novel delivery technologies, particularly targeted *in vivo* reprogramming via LNPs, could fundamentally disrupt the current centralized, complex, and costly ex vivo manufacturing model, potentially democratizing access to effective cellular immunotherapy. Ultimately, the goal is to evolve PSMA-CAR-T from an investigational modality into a cornerstone of curative-intent treatment for mCRPC. Achieving this will require sustained collaboration across academia, industry, and regulators, guided by the strategic, systems-level approach championed in this review.

## 7 Concluding remarks

The strategic path to establishing PSMA-CAR-T therapy as a cornerstone of curative-intent treatment for a defined subset of mCRPC patients, and a model for solid tumor CAR-T development, is now evident. We propose three interdependent, actionable priorities to galvanize the field:

First, a concerted, collaborative effort is required to create and maintain publicly accessible repositories of well-characterized, humanized mouse models engrafted with molecularly annotated mCRPC PDXs. These “living biobanks” must faithfully recapitulate key clinical challenges—heterogeneous PSMA expression, a human immune and stromal TME, and metastatic tropism. They will serve as an indispensable, reproducible platform for the rigorous preclinical validation of next-generation strategies before commitment to costly human trials.

Second, the era of conventional, small-scale trial design must end. Regulators, clinicians, and industry partners should collaborate to initiate adaptive platform trials (master protocols) for PSMA-CAR-T therapy in mCRPC. These trials should be mandatorily biomarker-enriched at entry and employ deep longitudinal biospecimen collection to generate predictive signatures of response and resistance. Adaptive designs will efficiently prune ineffective approaches and amplify promising ones, rapidly iterating toward optimized regimens.

Third, every clinical study must be conceived with a comprehensive, prospectively planned translational agenda that treats serial patient samples as a continuous data stream. The goal is to move beyond correlative analyses and build mechanistic, predictive models that disentangle the triad of response determinants: tumor-intrinsic factors (baseline and emergent genomic/antigenic landscape), TME context (spatial immune architecture, metabolic state), and CAR-T cell pharmacodynamics (expansion, trafficking, differentiation, exhaustion). Aggregating this knowledge across consortia will enable data-driven patient selection and therapy personalization.

In conclusion, while the challenges are formidable, the strategic path to navigating the current translational impasse is now clear. By embracing a collaborative, strategy-driven, and scientifically rigorous approach centered on the tripartite framework outlined in this review (**Figure 1**), and by harnessing the transformative potential of next-generation stem cell-derived immune effectors, the field can realize the curative potential of PSMA-CAR-T therapy for mCRPC and other solid malignancies.
